# Personalization of renal replacement therapy initiation: a secondary analysis of the AKIKI and IDEAL-ICU trials

**DOI:** 10.1186/s13054-022-03936-y

**Published:** 2022-03-21

**Authors:** François Grolleau, Raphaël Porcher, Saber Barbar, David Hajage, Abderrahmane Bourredjem, Jean-Pierre Quenot, Didier Dreyfuss, Stéphane Gaudry

**Affiliations:** 1 Centre of Research in Epidemiology and Statistics (CRESS), Université de Paris, French Institute of Health and Medical Research (INSERM U1153), French National Research Institute for Agriculture, Food, and Environment (INRAE), Paris, France; 2 Intensive Care Department, Nîmes University Hospital, University of Montpellier, Nîmes, France; 3 INSERM, Institut Pierre Louis d’Epidémiologie et de Santé Publique, AP-HP, Hôpital Pitié-Salpêtrière, Département de Santé Publique, Centre de Pharmacoépidémiologie, Sorbonne Université, Paris, France; 4 Clinical Epidemiology Unit, INSERM CIC1432, Dijon, and Clinical Investigation Center, Clinical Epidemiology/Clinical Trials Unit, Dijon Bourgogne University Hospital, Dijon, France; 5 Department of Intensive Care, François Mitterrand University Hospital, Lipness Team, INSERM Research Center, LNC-UMR1231 and LabEx LipSTIC, and INSERM CIC 1432, Clinical Epidemiology, University of Burgundy, Dijon, France; 6 Université de Paris, Service de Médecine Intensive-Réanimation, Hôpital Louis Mourier, AP-HP and INSERM, UMR S1155 “Common and Rare Kidney Diseases: From Molecular Events To Precision Medicine”, Sorbonne Université, Paris, France; 7 Service de Réanimation Médico-Chirurgicale, Hôpital Avicenne, APHP, UFR SMBH, Université Sorbonne Paris Nord, Bobigny, French National Institute of Health and Medical Research (INSERM), Common and Rare kidney Diseases (CORAKID), Hôpital Tenon, Paris, France

**Keywords:** Acute kidney injury, Renal replacement therapy, Heterogeneity of treatment effect, Personalized medicine

## Abstract

**Background:**

Trials comparing early and delayed strategies of renal replacement therapy in patients with severe acute kidney injury may have missed differences in survival as a result of mixing together patients at heterogeneous levels of risks. Our aim was to evaluate the heterogeneity of treatment effect on 60-day mortality from an early vs a delayed strategy across levels of risk for renal replacement therapy initiation under a delayed strategy.

**Methods:**

We used data from the AKIKI, and IDEAL-ICU randomized controlled trials to develop a multivariable logistic regression model for renal replacement therapy initiation within 48 h after allocation to a delayed strategy. We then used an interaction with spline terms in a Cox model to estimate treatment effects across the predicted risks of RRT initiation.

**Results:**

We analyzed data from 1107 patients (619 and 488 in the AKIKI and IDEAL-ICU trial respectively). In the pooled sample, we found evidence for heterogeneous treatment effects (*P* = 0.023). Patients at an intermediate-high risk of renal replacement therapy initiation within 48 h may have benefited from an early strategy (absolute risk difference, − 14%; 95% confidence interval, − 27% to − 1%). For other patients, we found no evidence of benefit from an early strategy of renal replacement therapy initiation but a trend for harm (absolute risk difference, 8%; 95% confidence interval, − 5% to 21% in patients at intermediate-low risk).

**Conclusions:**

We have identified a clinically sound heterogeneity of treatment effect of an early vs a delayed strategy of renal replacement therapy initiation that may reflect varying degrees of kidney demand-capacity mismatch.

**Supplementary Information:**

The online version contains supplementary material available at 10.1186/s13054-022-03936-y.

## Introduction

Acute kidney injury (AKI) affects approximately half of critically ill patients and is associated with high mortality and long-term sequelae [1]. Since its introduction in intensive care units (ICU) in the 1960s [2], renal replacement therapy (RRT) has proved to be a major breakthrough for the treatment of AKI, saving countless lives. However, the optimal timing for RRT initiation in patients with severe AKI has been controversial. This is illustrated by opposite hypotheses regarding which of an early or a delayed RRT initiation strategy would be superior to the other in the sample size calculation of recent multicenter randomized controlled trials (RCTs) [3–5]. Moreover, three trials—the largest on the tropic—did not demonstrate any survival benefit from either strategy over the other. Likewise, recent meta-analyses concluded that, in the absence of life-threatening condition, the timing of RRT initiation did not affect survival [6, 7].

One suggested reason for the lack of conclusive findings lies in the heterogeneous baseline characteristics of patients included in these trials [8]. Meaningful differences in survival may have been missed as a result of mixing together patients with potential benefit and potential harm from a given initiation strategy. For instance, one may hypothesize that an early RRT initiation strategy is harmful to the patients who would never start it under a delayed strategy. In fact, when a delayed strategy is implemented, we observed that between a third and half of the patients never met the criteria mandating RRT initiation. Conversely, experts have speculated that the patients who would be susceptible to benefit from an early initiation strategy are those who would initiate RRT within 48 h under a delayed strategy [9].

Patient management further tailored to individual’s characteristics is much anticipated in critical care medicine [10] and AKI [11]. In that respect, the conventional subgroup analyses performed “one variable at a time” fail to convey meaningful results as they cannot fully capture all the relevant heterogeneity in patient characteristics [12]. Conversely, approaches using multivariable models have the potential to address the challenge of heterogeneous treatment effects (HTE) [13].

The concept of kidney demand-capacity mismatch may be useful to the personalization of RRT initiation, but it has not been evaluated on robust clinical data [14]. In this study, we wished to test if estimating the degree of demand-capacity mismatch could guide RRT initiation strategies. We hypothesized that an early RRT initiation strategy is unnecessary or harmful to the patients at low risk of RRT initiation under a delayed strategy; and beneficial to the patients at a higher risk. Accordingly, we used data from two large multicenter RCTs on RRT timing to develop a risk prediction model for RRT initiation within 48 h after allocation to a delayed strategy and then estimated treatment effects within levels of predicted risks.

## Methods

### Ethical approval and research transparency

The AKIKI and the IDEAL-ICU trials received approval for all participating centers from competent French legal authority (Comité de Protection des Personnes d’Ile de France VI, ID RCB 2013-A00765-40, NCT01932190 for AKIKI and Comité de Protection des Personnes Est I ID RCB 2012-A00519-34 for IDEAL-ICU), and consent of patient or relatives was obtained before inclusion (except in emergencies where deferred consent was allowed by the Institutional Review Board). We transparently reported our analysis following the PATH [15] and TRIPOD [16] statements.

### Source of data

The study sample included participants from the AKIKI and IDEAL-ICU, two multicenter RCTs conducted in France. The AKIKI trial was conducted at 31 ICUs from September 2013 through January 2016 and recruited 619 patients with severe AKI who required mechanical ventilation, catecholamine infusion, or both (the vast majority with septic shock). The IDEAL-ICU trial recruited in 29 ICUs from July 2012 through October 2016 and included 488 patients with severe AKI and septic shock. Both trials randomly assigned (1:1) patients to either an early or a delayed strategy of RRT initiation. None of these trials showed a significant difference between the two strategies on 60-day mortality. The delayed strategy averted the need for RRT in 49% and 38% of patients in the AKIKI and IDEAL-ICU trials, respectively.

### Outcomes

The primary outcome of this study was death at day 60. Secondary outcomes included mean differences in number of days free of RRT, mechanical ventilation and intensive care at 28 days [17] across the same levels of risk.

### Prediction model development

We developed a risk prediction model for RRT initiation within 48 h after allocation to a delayed strategy. The derivation sample consisted of the 550 patients allocated to the delayed arms of the AKIKI (*n* = 308) and IDEAL-ICU (*n* = 242) trials (Table 1). We fit a logistic regression model, using predefined 14 predictors to predict the occurrence of RRT initiation within 48 h after the start of the delayed strategy. Candidate predictor variables were taken from the pre-randomization eligibility screening or clinical examination prior to randomization to the delayed strategy of RRT initiation and included age (years), gender (male vs female), potassium level (mmol/L), blood urea nitrogen level (mmol/L), pH (unitless), the ratio of creatinine at enrollment over creatinine at baseline (unitless), urine output (< 200 ml/day vs ≥ 200 ml/day, as was already categorized in the data), SOFA score at enrollment (unitless), weight (kg), heart failure (yes vs no), hypertension (yes vs no), diabetes mellitus (yes vs no), cirrhosis (yes vs no), non-corticosteroid immunosuppressive drug (yes vs no). Missing data were handled through multiple imputations by chained equations using outcomes as well as all aforementioned predictors in the imputation models [18]. Five independent imputed data sets were generated and analyzed separately. The nonlinearity of each continuous variable was assessed through penalized spline regression. All continuous variables appeared roughly linearly associated with the logit of the outcome probability; hence, no non-linear terms were used.

**Table 1:** Characteristics of the patients at randomization

Characteristic	Delayed strategy	Early strategy
	*n* = 550	*n* = 557
*Study*		
AKIKI	308 (56.0)	311 (55.8)
IDEAL-ICU	242 (44.0)	246 (44.2)
Age—year	67.7 (13.2)	66.5 (13.3)
Weight—kg	81.6 (22.2)	82.4 (22.2)
Male sex	352 (64.0)	351 (63.0)
*Pre-existing conditions*		
Heart failure	52 (9.5)	44 (7.9)
Hypertension	304 (55.3)	306 (54.9)
Diabetes mellitus	92 (16.7)	112 (20.1)
Cirrhosis	54 (9.8)	54 (9.7)
Respiratory disease	54 (9.8)	62 (11.1)
Cancer	100 (18.2)	91 (16.3)
Hemopathy	27 (4.9)	34 (6.1)
AIDS	2 (0.4)	5 (0.9)
Non-corticosteroid immunosuppressive drug	36 (6.5)	32 (5.7)
Organ graft	17 (3.1)	5 (0.9)
*Severity at enrollment*		
SOFA score (0 to 24)	11.5 (3.1)	11.4 (3.2)
Respiratory SOFA (0 to 4)	2.1 (1.1)	1.9 (1.1)
Hemodynamic SOFA (0 to 4)	3.5 (1.2)	3.5 (1.2)
Liver SOFA (0 to 4)	0.8 (1.1)	0.8 (1.1)
Coagulation SOFA (0 to 4)	2.1 (1.6)	2.2 (1.6)
Neurologic SOFA (0 to 4)	1.3 (1.5)	1.2 (1.5)
*Laboratory values*		
Baseline creatinine (IQR), μmol/L*	88 (71–97)	84 (71–97)
Creatinine at enrollment (IQR), μmol/L	268 (211–343)	267 (198–352)
Blood urea nitrogen at enrollment (IQR), mmol/L	19 (14–26)	19 (13–26)
Potassium at enrollment, mmol/L	4.4 (0.8)	4.4 (0.8)
Bicarbonate at enrollment, mmol/L	18 (5)	18 (5)
Arterial blood pH at enrollment	7.30 (0.10)	7.30 (0.10)

All characteristics reported in the table were determined at inclusion in the AKIKI or IDEAL-ICU trial, before initiation of renal replacement therapy

Data are mean (SD), median (IQR) or *n* (%). AIDS = Acquired Immunodeficiency Syndrome. IQR = Interquartile range. SOFA score = Sequential Organ Failure Assessment score

To convert the values for creatinine to milligrams per deciliter, divide by 88.4

*The serum creatinine concentration before ICU admission was either determined with the use of values measured in the 12 months preceding the ICU stay or was estimated

Two strategies were used to select predictors with the imputed data [19]. First, we used Wald tests for the pooled regression coefficients to simplify the model with a backward selection procedure, with *P-*value cut-offs mimicking the use of Akaike information criterion (e.g., a cut-off of 0.157 for variables with 1 *df*). We then used a conventional backward elimination procedure in each imputed data set and retained the model comprising the variables selected in most imputed data sets. Both strategies selected the same variables. Two-by-two interactions between each of the selected variables were then examined using Wald tests for the pooled regression coefficients. No higher-order interactions were considered. Regression coefficients estimates and their variances were then pooled across imputed data sets [20].

To evaluate the predictive ability of the model, we first calculated the apparent discrimination (c-statistic) and calibration (categorization by fifth of predicted risk) in the derivation sample. The c-statistic measures how well the model discriminates between the patients who initiated RRT within 48 h after allocation a delayed strategy and those who did not. The calibration curve, estimated using local regression [21]], contrasts observed vs predicted probabilities of events and evaluates the accuracy of the predictions. Internal validation of the model was performed by bootstrapping, which allows to correct regression coefficients and model performance for optimism [22]. The variable selection strategy was repeated in 200 bootstrap samples, and performance of models fit in each sample was evaluated in these samples and in the original sample. The differences between these two performances were averaged and taken as a measure of overoptimism. The c-statistic as well as the calibration intercept and slope were corrected for bias by subtracting measures of overoptimism to the apparent performance metrics.

### Risk categorization

In the AKIKI (*n* = 619), IDEAL-ICU (*n* = 488) and pooled (*n* = 1107) samples, we categorized patients by fifths of the risk predicted by our final model. In each fifth of risk, we compared early vs delayed strategy of RRT initiation on primary and secondary outcomes. To account for censoring, death at day 60 was calculated from the Kaplan–Meier estimator. As HTE are fundamentally a scale dependent concept [15], we evaluated treatment effects on the absolute risk difference and the hazard ratio scales. For each scale we computed a smooth curve of the treatment effect across levels of risks by using an interaction term between treatment arm and a two knots natural spline transform [23] of the predicted risk in a Cox model. We assessed the evidence for heterogeneous treatment effect by testing the null hypothesis that a Cox model using a linear interaction between treatment arm and the predicted risk fits data equally well as a Cox model using a similar interaction with a spline transform of the predicted risk [24]. Ninety-five percent confidence intervals (95% CI) were calculated by bootstrapping (1000 iterations). All analyses were performed using the R statistical software version 4.0.5 (The R Foundation). More precisely, we used the rms package for model building and internal validation, the survival package for survival analyses, the mgcv package for heterogeneous treatment effects assessment, the boot package for bootstrap, and the mice package for multiple imputation. For transparency and reproducibility, the computer code used in this study is available as an Additional file 1 at the Journal’s website.

## Results

### Prediction model for RRT initiation

**Fig. 1:**
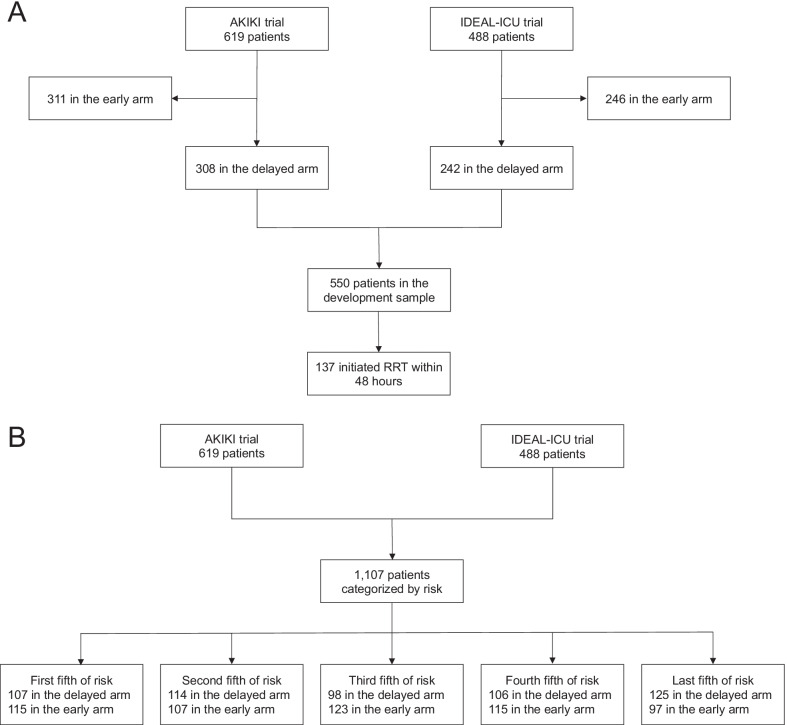
Study flow diagram for model development (Panel **A**) and the assessment of heterogeneous treatment effects across different levels of risk of RRT initiation within 48 hours after allocation to a delayed strategy (Panel **B**). RRT = Renal Replacement Therapy

Of the 550 patients included for model derivation (see Fig. 1, Panel A), 137 patients (25%) initiated RRT within 48 h after allocation to a delayed strategy (62 [20%] and 75 patients [31%] in AKIKI and IDEAL-ICU, respectively). 91% of patients had complete data for all candidate predictors (see Additional file 2: Figure S1); there were no missing data for the event of RRT initiation. The final prediction model included potassium, blood urea nitrogen, pH, non-corticosteroid immunosuppressive drug, SOFA and weight. No two-by-two interaction between variables was added as none showed statistical significance or seemed clinically informative.

**Fig. 2:**
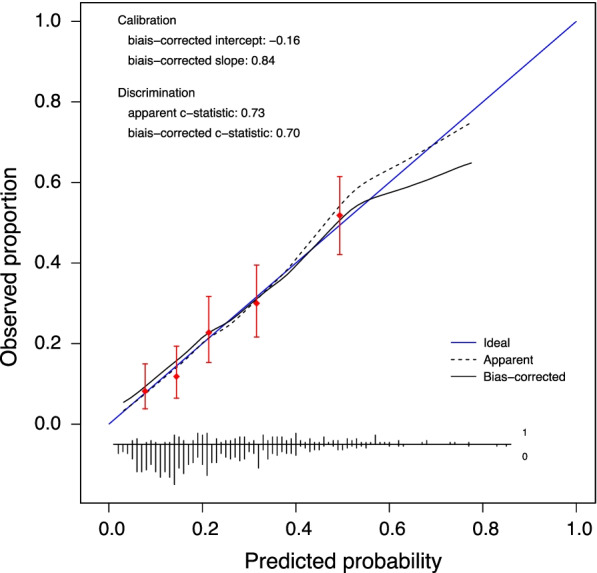
Internal calibration curve and performance of the final model for RRT initiation within 48 h after allocation to a delayed strategy. The blue line represents ideal calibration. Values of biased-corrected slope above 1 indicates underestimation of risks by the model; while values lower than 1 indicates overestimation. Non-corrected intercept and slope will be 0 and 1 by definition for the derivation sample

The full and final models are presented in Table 2. The apparent and bias-corrected c-statistic were 0.73 (95% CI: 0.70 to 0.80) and 0.70 (95% CI: 0.67 to 0.77), respectively. The predictive performance of the final model was good, as measured by discrimination and calibration (Fig. 2).

**Table 2:** Univariable analysis, full and final models for RRT initiation within 48 h after allocation to a delayed strategy

Variable	Univariable analysis				Full model				Final model			
	OR	(95% CI)		*P*	OR	(95% CI)		*P*	OR	(95% CI)		*P*
Age (year)	0.993	0.979	1.008	0.361	1.004	0.986	1.022	0.694	–	–	–	–
Sex (male vs female)	1.257	0.834	1.894	0.276	1.188	0.755	1.868	0.456	–	–	–	–
Potassium at enrollment (mmol/L)	1.728	1.343	2.224	< .001	1.381	1.048	1.821	0.022	1.391	1.057	1.831	0.019
Creatinine at enrollment over creatinine at baseline (unitless)	1.181	1.058	1.318	0.003	1.068	0.931	1.225	0.350	–	–	–	–
Urine output (< 200 ml/day vs > = 200 ml/day)	1.792	1.183	2.714	0.006	1.129	0.699	1.823	0.621	–	–	–	–
SOFA at enrollment (unitless)	1.151	1.079	1.227	< .001	1.123	1.041	1.210	0.003	1.139	1.063	1.221	< .001
Weight at enrollment (kg)	1.012	1.003	1.021	0.006	1.013	1.003	1.023	0.010	1.013	1.004	1.022	0.007
Heart failure (yes vs no)	0.792	0.395	1.588	0.512	0.845	0.399	1.788	0.659	–	–	–	–
Hypertension (yes vs no)	0.831	0.564	1.224	0.350	0.847	0.541	1.326	0.468	–	–	–	–
Diabetes mellitus (yes vs no)	1.153	0.695	1.913	0.582	0.985	0.558	1.741	0.960	–	–	–	–
Cirrhosis (yes vs no)	1.179	0.628	2.213	0.608	1.135	0.549	2.346	0.733	–	–	–	–
Non-corticosteroid immunosuppressive drug (yes vs no)	2.023	1.005	4.074	0.049	1.971	0.928	4.185	0.078	1.973	0.936	4.159	0.075
Blood urea nitrogen at enrollment (mmol/L)	1.029	1.009	1.050	0.004	1.022	0.998	1.047	0.076	1.026	1.004	1.048	0.021
pH at enrollment (OR for an increase of 0.01)	0.945	0.925	0.966	< .001	0.954	0.932	0.978	< .001	0.954	0.932	0.977	< .001

The intercepts were 1.35 × 10^12^ and 2.51 × 10^12^ for the full and final models respectively

### Heterogeneity of treatment effect

**Fig. 3:**
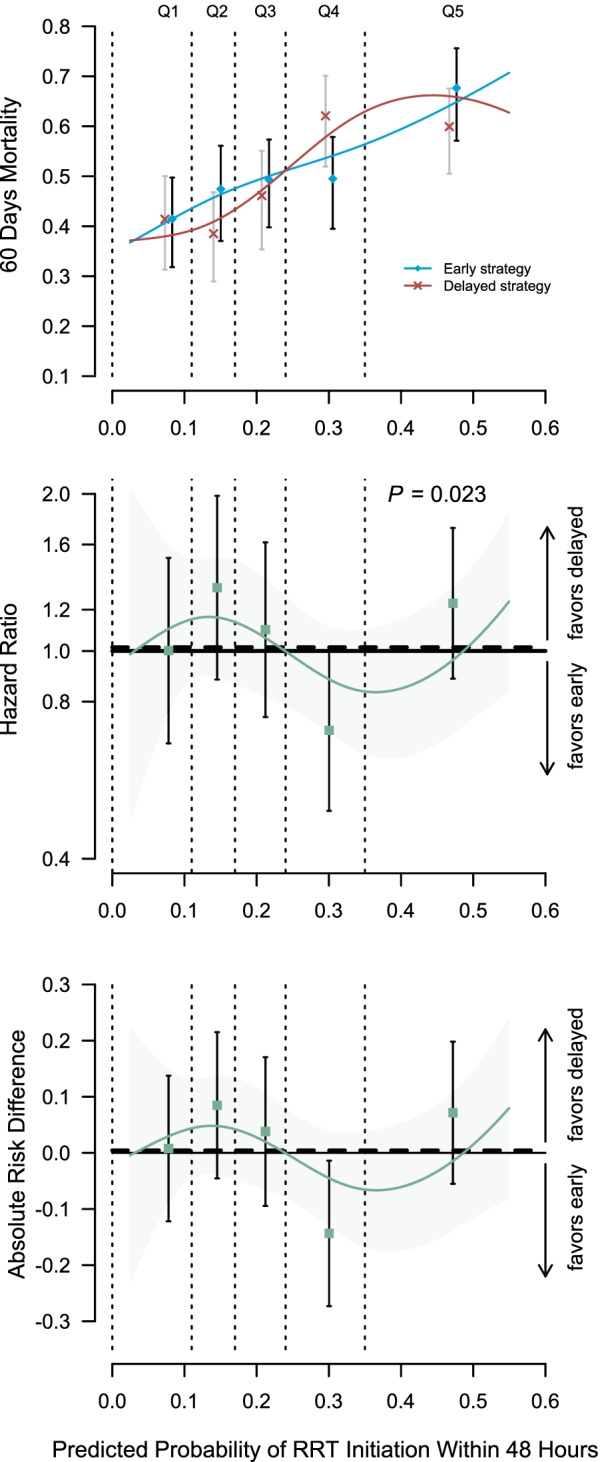
Heterogeneity of treatment effect (early vs delayed strategy) across different levels of risk of RRT initiation within 48 h after allocation to a delayed strategy. This figure presents heterogeneous treatment effect of an early vs a delayed strategy of RRT initiation as a function of the baseline risk of RRT initiation within 48 h after allocation to a delayed strategy in the pooled AKIKI and IDEAL-ICU sample. The horizontal dashed lines indicate the average treatment effect. *P* value for a constant effect along the predicted risk (test of heterogeneity of the treatment effect). Q1 = first fifth of risk (lowest), Q2 = second fifth of risk, Q3 = third fifth of risk, Q4 = fourth fifth of risk, Q5 = last fifth of risk (highest)

**Fig. 4:**
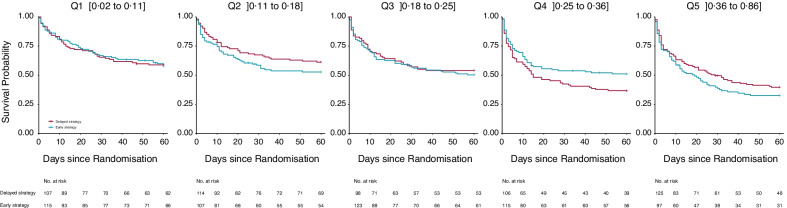
Kaplan–Meier Estimates of Survival at 60 days in each fifth of risk of RRT initiation within 48 h after allocation to a delayed strategy. Q1 = first fifth of risk (lowest), Q2 = second fifth of risk, Q3 = third fifth of risk, Q4 = fourth fifth of risk, Q5 = last fifth of risk (highest). Intervals corresponds to the minimal and maximal predicted probability of RRT initiation in each fifth

For the pooled AKIKI and IDEAL-ICU samples (*n* = 1,107 *see* Fig. 1, Panel B), baseline characteristics at randomization are presented in Table 1. In all fifth of risk predicted by our model, patients’ characteristics appeared balanced between the randomization arms (see Additional file 2: Table S1). Patients’ characteristics by fifth of risk predicted by our model are provided in the Additional file 2: Table S2. Heterogeneity of treatment effect is presented by fifth of risk in Fig. 3. There was no evidence of benefit from an early RRT initiation strategy for individuals within the lowest fifth of RRT initiation risk (absolute risk difference [ARD], 1%; 95% CI − 12% to 14%). However, patients in the fourth fifth of risk, may have benefited from an early strategy of RRT initiation (ARD, − 14%; 95% CI − 27% to − 1%). For patients with the highest risk (last fifth of risk), we found no evidence of benefit from an early initiation strategy (ARD, 7%; 95% CI − 6% to 20%). On both the absolute (i.e., ARD) and relative (i.e., event rate and hazard ratio) scales, the smooth curve suggested that an early RRT initiation strategy may be harmful in patients at an intermediate-low risk (second fifth of risk), while it may be beneficial in patients at an intermediate-high risk (fourth fifth of risk). This pattern was consistent in both the AKIKI and IDEAL-ICU trials when analyzed separately (*see* Additional file 2: Figure S2). Kaplan–Meier survival for each fifth of risk are given in Fig. 4 and in Additional file 2: Figure S3. No difference in secondary outcomes was found between early and delayed RRT initiation strategy in any fifth of predicted risk (*see* Additional file 2: Figure S4).

An implementation of our model has been made available via a user-friendly web interface at http://rrt-personalization.eu/**.** With this web application, clinicians and researchers can obtain the predicted probability of RRT initiation within 48 h after allocation to a delayed strategy in patients with severe AKI. The individual treatment effect of an early vs delayed strategy is then computed and returned with 95% CIs.

## Discussion

### Summary of findings

In this study, we developed a prediction model for the initiation of RRT within 48 h after allocation to a delayed strategy in patients with severe AKI in the ICU. We subsequently used the predictions from this model to identify subgroups (i.e., fifths) of patients at similar risk. We then assessed if the treatment effect of an early vs delayed strategy of RRT initiation was heterogeneous between these subgroups.

We stress that although causal understanding of model predictions is always inappropriate, in the case of the present HTE, this interpretation is proper as all variables included in our model were measured prior to randomization. In our main analysis, we found substantial HTE across levels of predicted risks. Except for the upper boundary (i.e., highest levels of risks), the directions of the HTE were aligned with our prespecified hypothesis.

From a clinical standpoint, the predicted risk from our model may be viewed as a proxy for the severity of kidney demand-capacity mismatch of the patients included in the trials. Through this lens, our results seem to indicate that for the most severe patients, an invasive strategy i.e., early RRT was unnecessary and/or harmful (ARD in the last fifth of predicted risk, 7%; 95% CI, − 6% to 20%). This seemed true also of mildly severe patients (ARD in the second fifth of predicted risk, 8%; 95% CI, − 5% to 21%). The only patients who seemed to have benefited from early RRT are those at a high but nonextreme risk (ARD in the fourth fifth of predicted risk, − 14%; 95% CI, − 27% to − 1%). An interpretation for these findings is that starting RRT early could harm the lesser severe patients because they often have no need for such invasive treatment. On the other hand, early RRT could be unnecessary to the most severe patients as their prognosis may outweigh potential benefits; or early RRT could even harm them through the destabilization of a weak equilibrium.

Hitherto, the concept of demand-capacity and personalization of RRT initiation did not rely on the analysis of robust clinical data. The 2021 Surviving Sepsis Campaign guidelines argues for a pragmatic approach: propose a wait-and-see strategy for all patients with severe AKI and no life-threatening complications in the intensive care unit [25].

### Strength and limitations

We acknowledge that given large enough sample sizes, more advanced machine learning techniques could potentially yield a more precise estimation of HTEs. These techniques, often referred to as effect-modelling approaches, aim to estimate HTE through direct modelling of the treatment effect [26]. Of note, they are also vulnerable to misspecification and overfitting, and therefore require huge sample sizes [27]. In contrast, we chose to implement a risk-modelling approach and relied on the PATH guidelines for personalized medicine [15]. On the upside, this allowed us to evaluate a clinically sound, a priori-specified hypothesis [9]. Compared to black-box algorithms, we believe the transparency of our parametric modelling methodology offers researchers a window for interpretability.

Despite the good performance of our prediction model as evaluated on biased-corrected metrics, the absence of external validation for our prediction model is a limitation. However, in our methodology, the model predictions are merely a mean for a downstream purpose namely, the assessment of HTEs. A poorly performing model would have limited our ability to find evidence of HTE when treatment effects are in fact truly heterogeneous.

Last, in contrast with other instances where predictions from developed models cannot be readily calculated by clinicians or researchers, we have implemented a user-friendly web interface for our approach. We trust this will help further disseminate, replicate, or refine our findings. We purposely chose to emphasize uncertainty for the individualized treatment effects by providing all metrics along with their 95% CI. We believe that as decision tools have not been evaluated in controlled settings, clinical judgment should however prevail.

### Implications for future research

Precision medicine is an active field of research with limited clinical applications so far [28]. Data-driven decision support tools have been made available in cardiology [29], while in critical care HTE were documented for crystalloid fluids [30] or ventilation strategies [31], In fact, as negative trial findings are widespread, disentangling HTE were judged a research priority in critical care [32]. The identification of HTE may also inform the design of adaptive trials [33]. For instance, enrichment trials recruiting only the patients most likely to benefit from an early RRT initiation strategy could yield larger treatment effect sizes [34].

We believe the risk-modelling methodology presented in our study is transportable to treatments as diverse as corticosteroids for sepsis [35], proton pump inhibitors for gastrointestinal bleeding prevention [36], or extracorporeal membrane oxygenation for acute respiratory distress syndrome [37].

As for RRT initiation strategies, our findings will require further replication using other data sources and methodologies. The way in which this can happen is twofold. First, as in the present study, researchers can consider the static case of an early vs delayed strategy of RRT initiation and use either other RCT data or observational data coupled with robust statistical methods. Second, researchers may also account for the fundamentally dynamic nature of the question. On the one hand, AKI staging systems inaccurately reflect the timing of the underlying pathology [38]; on the other hand definition of the criteria mandating RRT initiation under a delayed strategy ought to be refined [39, 40]. While the latter problem can be addressed with advanced causal inference techniques [41], the former can be tackled through cutting-edge pathophysiological studies. These two approaches are, in our view, complementary and we believe researchers should strive to dig from both ends.

In this secondary analysis of the AKIKI and IDEAL-ICU trials, we have provided proof-of-concept for the HTE of early vs delayed strategy across levels of baseline risk of RRT initiation within 48 h after a delayed strategy. Though consistent between the two trials, our results will require replication and refinement before they can be implemented in practice. We believe that the risk-modelling methodology we described can help move the precision medicine agenda forward as it may be applicable to a wide variety of treatments in critical care.

## Supplementary Information

**Additional file 1.** Computer code: analysis.R.

**Additional file 2.** **Table S1:** Characteristics of the patients at randomization in each arm by fifth of risk of RRT initiation within 48 hours after the start of a delayed strategy. **Table S2:** Characteristics of the patients at randomization by fifth of risk of RRT initiation within 48 hours after the start of a delayed strategy. **Figure S1:** Missing data in the delayed (Panel A) and early (Panel B) strategy arms. **Figure S2:** Heterogeneity of treatment effect (early vs delayed strategy) across different levels of risk of RRT initiation within 48 hours after allocation a delayed strategy in the AKIKI and IDEALICU samples. **Figure S3:** Kaplan-Meier estimates of survival at 60 days in each fifth of risk for the AKIKI, IDEAL-ICU and pooled samples. **Figure S4:** Results on secondary outcomes on the mean difference scale in each fifth of risk for the AKIKI, IDEAL-ICU and pooled samples.

## Data Availability

Anonymous participant data is available under specific conditions. Proposals will be reviewed and approved by the sponsor, scientific committee, and staff on the basis of scientific merit and absence of competing interests. Once the proposal has been approved, data can be transferred through a secure online platform after the signing of a data access agreement and a confidentiality agreement.

## Abbreviations

AKI: Acute Kidney Injury
ARD: Absolute Risk Difference
CI: Confidence Interval
HTE: Heterogeneous Treatment Effects
HR: Hazard Ratio
ICU: Intensive Care Unit
PATH: Predictive Approaches to Treatment effect Heterogeneity
RCT: Randomized Controlled Trial
RRT: Renal Replacement Therapy
SOFA: Sequential Organ Failure Assessment
TRIPOD: Transparent Reporting of a multivariable prediction model for Individual Prognosis Or Diagnosis
