# Supplementary material for: Personalization of renal replacement therapy initiation: a secondary analysis of the AKIKI and IDEAL-ICU trials
Source: Crit Care. 2022 Mar 21;26:64. doi: 10.1186/s13054-022-03936-y (PMC8939225; doi:10.1186/s13054-022-03936-y)
Supplement: Supplementary file 2 — Additional file 2. Table S1: Characteristics of the patients at randomization in each arm by fifth of risk of RRT initiation within 48 hours after the start of a delayed strategy. Table S2: Characteristics of the patients at randomization by fifth of risk of RRT initiation within 48 hours after the start of a delayed strategy. Figure S1: Missing data in the delayed (Panel A) and early (Panel B) strategy arms. Figure S2: Heterogeneity of treatment effect (early vs delayed strategy) across different levels of risk of RRT initiation within 48 hours after allocation a delayed strategy in the AKIKI and IDEALICU samples. Figure S3: Kaplan-Meier estimates of survival at 60 days in each fifth of risk for the AKIKI, IDEAL-ICU and pooled samples. Figure S4: Results on secondary outcomes on the mean difference scale in each fifth of risk for the AKIKI, IDEAL-ICU and pooled samples. [file 13054_2022_3936_MOESM2_ESM.docx]

**Personalization of renal replacement therapy initiation: a secondary analysis of the AKIKI and IDEAL-ICU trials**

François GROLLEAU,^1^ Raphaël PORCHER,^2^ Saber BARBAR,^3^ David HAJAGE,^4^ Abderrahmane BOURREDJEM,^5^ Jean-Pierre QUENOT*,^6^ Didier DREYFUSS*,^7^ Stéphane GAUDRY*.^8^

* These authors contributed equally as senior authors.

ADITIONAL FILE 1

**Table of Contents**

[Supplementary Results 3](#_Toc95573945)

[Table S1. Characteristics of the patients at randomization in each arm by fifth of risk of RRT initiation within 48 hours after the start of a delayed strategy. 3](#_Toc95573946)

[Table S2. Characteristics of the patients at randomization by fifth of risk of RRT initiation within 48 hours after the start of a delayed strategy. 4](#_Toc95573947)

[Figure S1. Missing data in the delayed (Panel A) and early (Panel B) strategy arms. 5](#_Toc95573948)

[Figure S2. Heterogeneity of treatment effect (early vs delayed strategy) across different levels of risk of RRT initiation within 48 hours after allocation a delayed strategy in the AKIKI and IDEAL-ICU samples. 6](#_Toc95573949)

[Figure S3. Kaplan-Meier estimates of survival at 60 days in each fifth of risk for the AKIKI, IDEAL-ICU and pooled samples. 7](#_Toc95573950)

[Figure S4. Results on secondary outcomes on the mean difference scale in each fifth of risk for the AKIKI, IDEAL-ICU and pooled samples. 8](#_Toc95573951)

# Supplementary Results

## Table S1. Characteristics of the patients at randomization in each arm by fifth of risk of RRT initiation within 48 hours after the start of a delayed strategy.

All characteristics reported in the table were determined at inclusion in the AKIKI or IDEAL-ICU trial, before initiation of renal replacement therapy. Intervals corresponds to the minimal and maximal predicted probability of RRT initiation in each fifth. *P* values are not adjusted for multiplicity.

|  | **Q1 [0.0211,0.114]** | |  | **Q2 (0.114,0.177]** | |  | **Q3 (0.177,0.249]** | |  | **Q4 (0.249,0.355]** | |  | **Q5 (0.355,0.862]** | |  |
| --- | --- | --- | --- | --- | --- | --- | --- | --- | --- | --- | --- | --- | --- | --- | --- |
|  | Delayed strategy | Early strategy | *P* | Delayed strategy | Early strategy | *P* | Delayed strategy | Early strategy | *P* | Delayed strategy | Early strategy | *P* | Delayed strategy | Early strategy | *P* |
| **Characteristic** | n=107 | n=115 |  | n=114 | n=107 |  | n=98 | n=123 |  | n=106 | n=115 |  | n=125 | n=97 |  |
| Study |  |  |  |  |  |  |  |  |  |  |  |  |  |  |  |
|  |  |  |  |  |  |  |  |  |  |  |  |  |  |  |  |
| IDEAL-ICU | 32 (29.9) | 39 (33.9) | 0.620 | 44 (38.6) | 40 (37.4) | 0.962 | 40 (40.8) | 64 (52.0) | 0.128 | 57 (53.8) | 51 (44.3) | 0.206 | 69 (55.2) | 52 (53.6) | 0.920 |
| Age — year | 70.40 (12.53) | 65.66 (14.76) | 0.011 | 65.91 (15.09) | 66.03 (14.33) | 0.955 | 69.55 (12.11) | 67.68 (12.22) | 0.257 | 67.21 (13.75) | 67.96 (11.81) | 0.662 | 65.84 (11.80) | 65.02 (13.44) | 0.627 |
| Weight — kg | 72.29 (17.73) | 73.02 (14.39) | 0.735 | 77.24 (17.27) | 78.01 (16.31) | 0.735 | 82.77 (18.58) | 83.12 (21.29) | 0.896 | 83.15 (21.13) | 84.77 (20.35) | 0.560 | 91.88 (28.31) | 94.67 (30.95) | 0.485 |
| Male sex | 65 (60.7) | 62 (53.9) | 0.372 | 65 (57.0) | 71 (66.4) | 0.198 | 64 (65.3) | 73 (59.3) | 0.443 | 75 (70.8) | 81 (70.4) | 1.000 | 83 (66.4) | 64 (66.0) | 1.000 |
| Pre-existing conditions |  |  |  |  |  |  |  |  |  |  |  |  |  |  |  |
| Heart failure | 11 (10.3) | 10 (8.7) | 0.862 | 10 (8.8) | 7 (6.5) | 0.712 | 8 (8.2) | 6 (4.9) | 0.473 | 12 (11.3) | 5 (4.3) | 0.091 | 11 (8.8) | 16 (16.5) | 0.125 |
| Hypertension | 64 (59.8) | 56 (48.7) | 0.127 | 62 (54.4) | 57 (53.3) | 0.975 | 57 (58.2) | 74 (60.2) | 0.871 | 56 (52.8) | 60 (52.2) | 1.000 | 65 (52.0) | 59 (60.8) | 0.239 |
| Diabetes mellitus | 15 (14.0) | 16 (13.9) | 1.000 | 18 (15.8) | 23 (21.5) | 0.359 | 11 (11.2) | 28 (22.8) | 0.040 | 20 (18.9) | 22 (19.1) | 1.000 | 28 (22.4) | 23 (23.7) | 0.945 |
| Cirrhosis | 10 (9.3) | 9 (7.8) | 0.869 | 10 (8.8) | 6 (5.6) | 0.517 | 7 (7.1) | 13 (10.6) | 0.518 | 12 (11.3) | 10 (8.7) | 0.670 | 15 (12.0) | 16 (16.5) | 0.445 |
| Respiratory Disease | 9 (8.4) | 12 (10.4) | 0.775 | 14 (12.3) | 16 (15.0) | 0.702 | 14 (14.3) | 10 (8.1) | 0.214 | 7 (6.6) | 11 (9.6) | 0.577 | 10 (8.0) | 13 (13.4) | 0.277 |
| Cancer | 22 (20.6) | 22 (19.1) | 0.921 | 19 (16.7) | 15 (14.0) | 0.720 | 18 (18.4) | 17 (13.8) | 0.463 | 18 (17.0) | 20 (17.4) | 1.000 | 23 (18.4) | 17 (17.5) | 1.000 |
| Hemopathy | 7 (6.5) | 8 (7.0) | 1.000 | 3 (2.6) | 5 (4.7) | 0.652 | 4 (4.1) | 6 (4.9) | 1.000 | 3 (2.8) | 10 (8.7) | 0.118 | 10 (8.0) | 5 (5.2) | 0.570 |
| AIDS | 0 (0.0) | 1 (0.9) | 1.000 | 2 (1.8) | 1 (0.9) | 1.000 | 0 (0.0) | 1 (0.8) | 1.000 | 0 (0.0) | 0 (0.0) | — | 0 (0.0) | 2 (2.1) | 0.370 |
| Non-corticosteroid immunosuppressive drug | 2 (1.9) | 1 (0.9) | 0.950 | 1 (0.9) | 1 (0.9) | 1.000 | 6 (6.1) | 6 (4.9) | 0.915 | 6 (5.7) | 12 (10.4) | 0.294 | 21 (16.8) | 12 (12.4) | 0.465 |
| Organ graft | 1 (0.9) | 0 (0.0) | 0.971 | 1 (0.9) | 1 (0.9) | 1.000 | 3 (3.1) | 1 (0.8) | 0.461 | 2 (1.9) | 2 (1.7) | 1.000 | 10 (8.0) | 1 (1.0) | 0.039 |
| Severity at enrollment |  |  |  |  |  |  |  |  |  |  |  |  |  |  |  |
| SOFA score (0 to 24) | 8.59 (2.39) | 8.50 (2.62) | 0.783 | 10.68 (2.46) | 10.78 (2.61) | 0.789 | 11.11 (2.28) | 11.69 (2.58) | 0.083 | 13.12 (2.60) | 12.58 (2.65) | 0.128 | 13.56 (2.86) | 13.76 (2.82) | 0.598 |
| Respiratory SOFA (0 to 4) | 2.18 (1.17) | 1.88 (1.09) | 0.050 | 2.03 (1.03) | 1.96 (1.16) | 0.665 | 2.05 (1.06) | 1.85 (1.16) | 0.194 | 1.95 (1.22) | 1.94 (1.18) | 0.933 | 2.18 (1.25) | 2.00 (1.19) | 0.289 |
| Hemodynamic SOFA (0 to 4) | 2.82 (1.65) | 2.63 (1.71) | 0.407 | 3.28 (1.40) | 3.51 (1.14) | 0.179 | 3.49 (1.17) | 3.63 (1.01) | 0.326 | 3.76 (0.76) | 3.69 (0.99) | 0.518 | 3.78 (0.78) | 3.88 (0.60) | 0.335 |
| Liver SOFA (0 to 4) | 0.56 (0.87) | 0.54 (0.88) | 0.854 | 0.74 (0.93) | 0.64 (0.99) | 0.478 | 0.67 (0.93) | 0.87 (1.09) | 0.158 | 0.94 (1.09) | 0.77 (1.10) | 0.253 | 1.18 (1.26) | 1.14 (1.30) | 0.819 |
| Coagulation SOFA (0 to 4) | 2.57 (1.58) | 2.43 (1.74) | 0.547 | 2.19 (1.61) | 2.50 (1.54) | 0.156 | 2.10 (1.66) | 1.91 (1.60) | 0.386 | 1.88 (1.54) | 2.03 (1.47) | 0.438 | 1.82 (1.53) | 1.99 (1.50) | 0.398 |
| Neurologic SOFA (0 to 4) | 0.74 (1.27) | 0.92 (1.32) | 0.293 | 1.19 (1.44) | 1.14 (1.51) | 0.791 | 1.22 (1.51) | 1.14 (1.39) | 0.659 | 1.69 (1.61) | 1.41 (1.59) | 0.195 | 1.57 (1.50) | 1.65 (1.61) | 0.698 |
| Laboratory values |  |  |  |  |  |  |  |  |  |  |  |  |  |  |  |
| Baseline creatinine, μmol/L* | 82.68 (24.92) | 75.44 (22.30) | 0.023 | 83.33 (24.43) | 83.98 (26.91) | 0.850 | 89.28 (30.07) | 84.43 (26.64) | 0.206 | 96.72 (44.31) | 85.04 (32.90) | 0.026 | 93.69 (45.76) | 103.85 (51.06) | 0.120 |
| Creatinine at enrollment, μmol/L | 235.10 (107.57) | 228.40 (93.64) | 0.620 | 269.88 (101.47) | 269.40 (107.60) | 0.973 | 284.28 (107.21) | 295.82 (126.79) | 0.473 | 311.57 (124.28) | 319.94 (148.00) | 0.651 | 342.49 (156.06) | 317.32 (129.46) | 0.201 |
| Blood urea nitrogen at enrollment, mmol/L | 15.99 (6.92) | 15.81 (7.29) | 0.846 | 18.73 (9.41) | 18.29 (7.37) | 0.697 | 20.51 (7.44) | 20.28 (9.04) | 0.844 | 22.28 (9.89) | 21.76 (10.25) | 0.701 | 25.30 (10.81) | 23.41 (9.24) | 0.171 |
| Potassium at enrollment, mmol/L | 3.89 (0.55) | 3.94 (0.65) | 0.564 | 4.17 (0.62) | 4.22 (0.75) | 0.588 | 4.46 (0.63) | 4.37 (0.64) | 0.338 | 4.46 (0.73) | 4.55 (0.67) | 0.345 | 5.06 (0.81) | 4.86 (0.84) | 0.071 |
| Bicarbonate at enrollment, mmol/L | 21.20 (5.38) | 20.25 (5.19) | 0.186 | 18.24 (4.18) | 18.93 (5.40) | 0.287 | 18.28 (5.59) | 17.39 (4.81) | 0.205 | 17.12 (4.28) | 17.84 (4.34) | 0.218 | 16.91 (4.94) | 16.74 (4.65) | 0.797 |
| Arterial blood pH at enrollment | 7.39 (0.07) | 7.39 (0.07) | 0.467 | 7.33 (0.07) | 7.34 (0.08) | 0.320 | 7.29 (0.07) | 7.31 (0.07) | 0.161 | 7.27 (0.07) | 7.27 (0.07) | 0.958 | 7.22 (0.10) | 7.20 (0.08) | 0.233 |
| Data are mean (SD) or n (%); AIDS=Acquired Immunodeficiency Syndrome; SOFA score=Sequential Organ Failure Assessment score.  * The serum creatinine concentration before ICU admission was either determined with the use of values measured in the 12 months preceding the ICU stay or was estimated. To convert the values for creatinine to milligrams per deciliter, divide by 88.4. | | | | | | | | | | | | | | | |

## Table S2. Characteristics of the patients at randomization by fifth of risk of RRT initiation within 48 hours after the start of a delayed strategy.

All characteristics reported in the table were determined at inclusion in the AKIKI or IDEAL-ICU trial, before initiation of renal replacement therapy. Intervals corresponds to the minimal and maximal predicted probability of RRT initiation in each fifth. *P* values are not adjusted for multiplicity.

| **Characteristic** | **Q1** | **Q2** | **Q3** | **Q4** | **Q5** | *P* |
| --- | --- | --- | --- | --- | --- | --- |
|  | [0.0211,0.114] | (0.114,0.177] | (0.177,0.249] | (0.249,0.355] | (0.355,0.862] |  |
|  | n=222 | n=221 | n=221 | n=221 | n=222 |  |
| Study |  |  |  |  |  |  |
| AKIKI | 151 (68.0) | 137 (62.0) | 117 (52.9) | 113 (51.1) | 101 (45.5) |  |
| IDEAL-ICU | 71 (32.0) | 84 (38.0) | 104 (47.1) | 108 (48.9) | 121 (54.5) | <0.001 |
| Age — year | 67.9 (13.9) | 65.9 (14.7) | 68.5 (12.2) | 67.6 (12.7) | 65.5 (12.5) | 0.072 |
| Weight — kg | 72.7 (16.0) | 77.6 (16.8) | 82.9 (20.1) | 84.0 (20.7) | 93.1 (29.5) | <0.001 |
| Male sex | 127 (57.2) | 136 (61.5) | 137 (62.0) | 156 (70.6) | 147 (66.2) | 0.043 |
| Pre-existing conditions |  |  |  |  |  |  |
| Heart failure | 21 (9.5) | 17 (7.7) | 14 (6.3) | 17 (7.7) | 27 (12.2) | 0.227 |
| Hypertension | 120 (54.1) | 119 (53.8) | 131 (59.3) | 116 (52.5) | 124 (55.9) | 0.652 |
| Diabetes mellitus | 31 (14.0) | 41 (18.6) | 39 (17.6) | 42 (19.0) | 51 (23.0) | 0.189 |
| Cirrhosis | 19 (8.6) | 16 (7.2) | 20 (9.0) | 22 (10.0) | 31 (14.0) | 0.162 |
| Respiratory Disease | 21 (9.5) | 30 (13.6) | 24 (10.9) | 18 (8.1) | 23 (10.4) | 0.430 |
| Cancer | 44 (19.8) | 34 (15.4) | 35 (15.8) | 38 (17.2) | 40 (18.0) | 0.742 |
| Hemopathy | 15 (6.8) | 8 (3.6) | 10 (4.5) | 13 (5.9) | 15 (6.8) | 0.507 |
| AIDS | 1 (0.5) | 3 (1.4) | 1 (0.5) | 0 (0.0) | 2 (0.9) | 0.442 |
| Non-corticosteroid immunosuppressive drug | 3 (1.4) | 2 (0.9) | 12 (5.4) | 18 (8.1) | 33 (14.9) | <0.001 |
| Organ graft | 1 (0.5) | 2 (0.9) | 4 (1.8) | 4 (1.8) | 11 (5.0) | 0.007 |
| Severity at enrollment |  |  |  |  |  |  |
| SOFA score (0 to 24) | 8.5 (2.5) | 10.7 (2.5) | 11.4 (2.5) | 12.8 (2.6) | 13.6 (2.8) | <0.001 |
| Respiratory SOFA (0 to 4) | 2.0 (1.1) | 2.0 (1.1) | 1.9 (1.1) | 1.9 (1.2) | 2.1 (1.2) | 0.595 |
| Hemodynamic SOFA (0 to 4) | 2.7 (1.7) | 3.4 (1.3) | 3.6 (1.1) | 3.7 (0.9) | 3.8 (0.7) | <0.001 |
| Liver SOFA (0 to 4) | 0.5 (0.9) | 0.7 (1.0) | 0.8 (1.0) | 0.9 (1.1) | 1.2 (1.3) | <0.001 |
| Coagulation SOFA (0 to 4) | 2.5 (1.7) | 2.3 (1.6) | 2.0 (1.6) | 1.9 (1.5) | 1.8 (1.5) | <0.001 |
| Neurologic SOFA (0 to 4) | 0.8 (1.3) | 1.2 (1.5) | 1.2 (1.4) | 1.5 (1.6) | 1.6 (1.5) | <0.001 |
| Laboratory values |  |  |  |  |  |  |
| Baseline creatinine, μmol/L* | 79 (23) | 84 (26) | 85 (28) | 90 (39) | 98 (48) | <0.001 |
| Creatinine at enrollment, μmol/L | 232 (100) | 269 (104) | 291 (118) | 316 (137) | 331 (145) | <0.001 |
| Blood urea nitrogen at enrollment, mmol/L | 16 (7) | 19 (8) | 20 (8) | 22 (10) | 24 (10) | <0.001 |
| Potassium at enrollment, mmol/L | 3.9 (0.6) | 4.2 (0.7) | 4.4 (0.6) | 4.5 (0.7) | 5.0 (0.8) | <0.001 |
| Bicarbonate at enrollment, mmol/L | 21 (5) | 19 (5) | 18 (5) | 17 (4) | 17 (5) | <0.001 |
| Arterial blood pH at enrollment | 7.39 (0.07) | 7.34 (0.07) | 7.30 (0.07) | 7.27 (0.07) | 7.21 (0.09) | <0.001 |

Data are mean (SD) or n (%); AIDS=Acquired Immunodeficiency Syndrome; SOFA score=Sequential Organ Failure Assessment score.

* The serum creatinine concentration before ICU admission was either determined with the use of values measured in the 12 months preceding the ICU stay or was estimated. To convert the values for creatinine to milligrams per deciliter, divide by 88.4.

## Figure S1. Missing data in the delayed (Panel A) and early (Panel B) strategy arms.

The tables show all missing data patterns observed. Vertical bars show the number of patients corresponding to the missing data pattern underneath. Horizontal bars show the number of patients with missing data corresponding to the variable aside. 500 patients (91%) had complete data with no missing candidate predictors in the delayed arms. 501 patients (90%) had complete data with no missing candidate predictors in the early arms. BUN=Blood Urea Nitrogen.

## Figure S2. Heterogeneity of treatment effect (early vs delayed strategy) across different levels of risk of RRT initiation within 48 hours after allocation a delayed strategy in the AKIKI and IDEAL-ICU samples.

This figure presents the heterogenous of treatment effects of an early vs a delayed strategy of RRT initiation as a function of the baseline risk of RRT initiation within 48 hours after a delayed strategy. The dashed lines indicate the average treatment effects in the corresponding sample. Q1 = first fifth of risk (lowest), Q2 = second fifth of risk, Q3 = third fifth of risk, Q4 = fourth fifth of risk, Q5 = last fifth of risk (highest).

## Figure S3. Kaplan-Meier estimates of survival at 60 days in each fifth of risk for the AKIKI, IDEAL-ICU and pooled samples.

Q1 = first fifth of risk (lowest), Q2 = second fifth of risk, Q3 = third fifth of risk, Q4 = fourth fifth of risk, Q5 = last fifth of risk (highest). Kaplan-Meier curves on the left-hand side are identical to those depicted in Figure 3 of the main text.

## Figure S4. Results on secondary outcomes on the mean difference scale in each fifth of risk for the AKIKI, IDEAL-ICU and pooled samples.

Q1 = first fifth of risk (lowest), Q2 = second fifth of risk, Q3 = third fifth of risk, Q4 = fourth fifth of risk, Q5 = last fifth of risk (highest).
